# Serotonin and catecholaminergic polymorphic ventricular tachycardia: a possible therapeutic role for SSRI s?

**DOI:** 10.5830/CVJA-2010-023

**Published:** 2010-08

**Authors:** SHUNJUAN CHEN, KAI TANG, DONGDONG ZHAO, YAWEI XU, QIANGLIN DUAN

**Affiliations:** Department of Cardiology, 10th People’s Hospital of Tongji University, Shanghai, China; Department of Cardiology, 10th People’s Hospital of Tongji University, Shanghai, China; Department of Cardiology, 10th People’s Hospital of Tongji University, Shanghai, China; Department of Cardiology, 10th People’s Hospital of Tongji University, Shanghai, China; Department of Scientific Research, Tongji Hospital of Tongji University, Shanghai, China

**Keywords:** catecholamine, polymorphi, ventricular tachycardia, gene mutation, selective serotonin reuptake inhibitor

## Abstract

Catecholaminergic polymorphic ventricular tachycardia (CPVT) is a rare malignant arrhythmia, usually diagnosed in the adolescent years. The diagnosis can typically be made by one or more of the following: a positive family history, exercise electrocardiography, ambulatory ECG monitoring and/ or an intra-cardiac, electrophysiological examination. This is a case report of a patient with CPVT that was refractory to treatment with beta-blockade and an implanted automatic cardioverter defibrillator. However, after a selective serotonin re-uptake inhibitor (SSRI) was added to the therapeutic regimen, no further episodes of ventricular tachycardia occurred during the following two years.

## Introduction

Catecholaminergic polymorphic ventricular tachycardia (CPVT) is one of the malignant ventricular arrhythmias, which was first reported by Leenhardt in 1995. CPVT is a familial disease with genetic mutations detectable in approximately 50% of patients. These include the RyR2 (ryanodine receptor) and CASQ2 (calsequestrin) mutations.^1^ CPVT often presents during childhood and the adolescent years, with the main clinical manifestation of sudden syncope during exercise or episodes of emotional distress. During such episodes there are often no known triggers present, such as electrolyte disturbances. It is important to exclude one of the primary ion channel diseases, such as long- QT syndrome (LQTS) and Brugada syndrome.

## Case report

A 23-year-old woman presented to our clinic with the clinical problem of repeated syncope events over the past 15 years. These attacks were more frequent during episodes of emotional distress.

During 2004, 24-hour ambulatory ECG monitoring was done by the referring hospital. It revealed frequent episodes of multifocal ventricular premature contractions. Because of this, metoprolol (12.5 mg twice daily) was prescribed. The patient was also sent for a psychological evaluation, and anxiety disorder with panic attacks was diagnosed and, subsequently, paroxetine (20 mg daily) was also prescribed.

Thereafter the patient was asymptomatic with no further syncope attacks for a period of a year. Because of the asymptomatic period, the patient stopped the metoprolol and paroxetine without medical consultation in 2005.

**Fig. 1.**
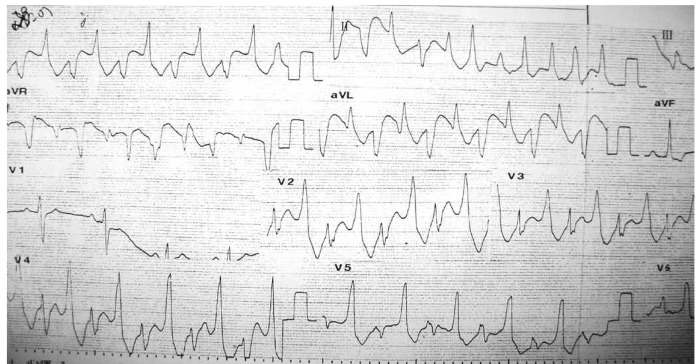
12-lead ECG of patient's ventricular tachycardia.

**Fig. 2.**
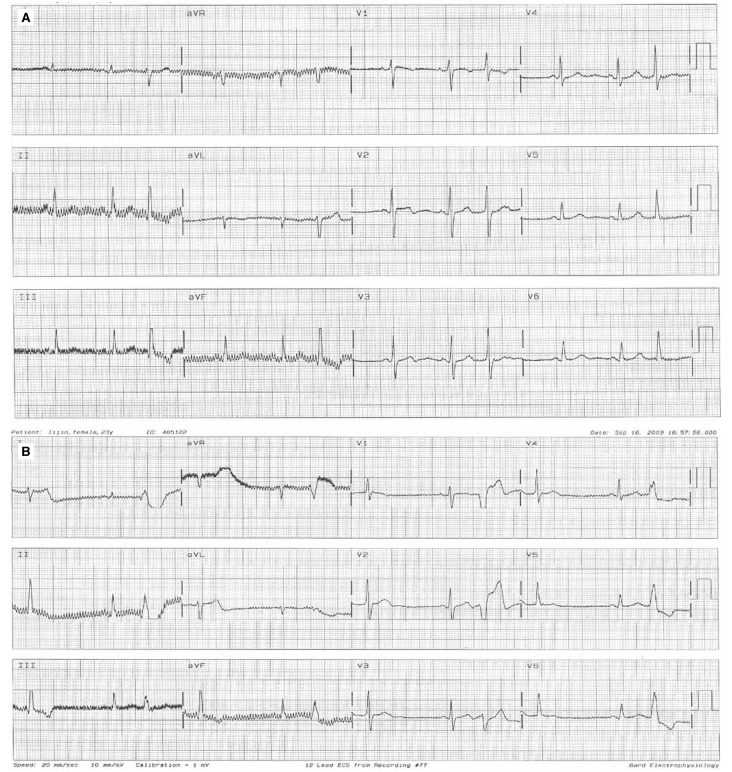
Regular ECG after admission. A: ventricular beats, B: supraventricular ectopic beats.

Two weeks before admission to our hospital, the episodes of syncope reappeared more intensely than before. Electrocardiography on admission demonstrated non-torsade de pointes ventricular tachycardia (Fig. 1). The regular ECG after admission showed ventricular ectopic beats (Fig. 2A) and supraventricular ectopic beats (Fig. 2B). Subsequent ambulatory ECG monitoring (22 hours and 53 min) after hospitalisation demonstrated a ventricular rate varying from 101 to 40 beats per minute and frequent polymorphic premature ventricular contractions with 416 episodes per hour.

A comprehensive biochemical evaluation, which included a coagulation profile, liver and kidney functions, electrolytes, glucose, auto-antibodies, thyroid hormone levels and thyroid antibodies, and antibodies to Coxsackie virus, were all within normal limits. Resting echocardiography showed normal cardiac structure and function, mild mitral regurgitation, the left atrial dimension was 38 mm (25–40 mm), interventricular septum (IVS) was 11 mm (6–11 mm), left ventricular dimensions (LVIDd and LVIDs) were 45 mm (37–56 mm) and 30 mm (23–35 mm), and left ventricular ejection fraction (LVEF) was 64% (> 50%).

After admission, the patient was treated with potassium and magnesium supplementation and beta-blocker therapy. During an intra-cardiac electrophysiological examination, repeated right atrial and ventricular stimulation did not evoke any atrial arrhythmia, ventricular tachycardia or ventricular fibrillation.

**Fig. 3.**
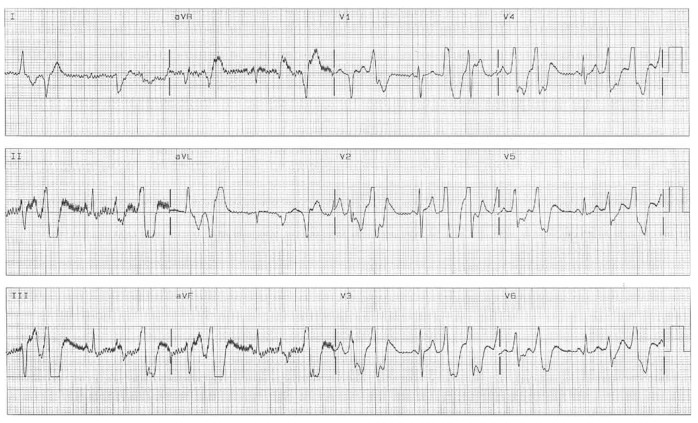
Premature ventricular contractions and short episodes of PVT occurred after intravenous infusion of isoproterenol.

Whenever the ventricular rate increased to more than 100 beats/min, frequent premature ventricular contractions and short episodes of polymorphic ventricular tachycardia were observed (Fig. 3). All of these episodes terminated spontaneously after a few minutes. The diagnosis of CPVT was made.

**Fig. 4.**
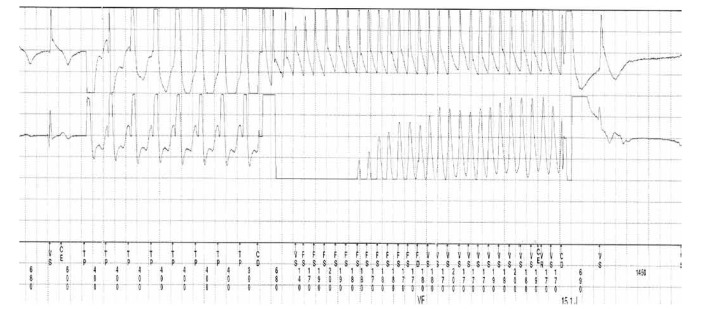
ICD discharges.

It was decided to perform ICD implantation without radiofrequency ablation. Postoperatively, the patient continued taking a beta-blocker orally. Six months after ICD implantation, pacemaker programming revealed two appropriate ICD discharges (Fig. 4). As the patient had previously been diagnosed with anxiety disorder and panic attacks, Seroxat (SSRI) 20 mg daily was added, and during a two-year follow-up period, no further ICD discharges occurred.

## Discussion

CPVT is an arrhythmic disorder with a high fatality rate. The incidence of adverse events, including syncope, ventricular tachycardia and ventricular fibrillation at 40 years of age is about 80%,^2^ and between 20 and 30 years, the incidence of sudden cardiac death is 30 to 50%.3 Currently, there are two known genetic mutations in CPVT, namely, RyR2 and CASQ2.^4^ These two ion channels mediate the transportation of calcium ions from the sarcoplasmic reticulum into the cytoplasm. Mutations in these two genes lead to intracellular calcium overload as the basis for the subsequent arrhythmias.

Sympathetic stimulation may lead to delayed after-depolarisations (DAD) and triggered activity, which will also induce arrhythmias. Cerrone et al.^5^ demonstrated that anesthetised mice with RyR2 mutations had a higher incidence of CPVT than in the isolated heart, suggesting that sympathetic stimulation may have an impact on CPVT.

Beta-blockers are the first-line agents for the treatment of CPVT. They are used to prevent episodes of ventricular tachycardia. However, the efficacy of beta-blockers in CPVT is inferior when compared to LQT1 syndrome (KCNQ1 gene mutation), but is comparable to the LQT2 syndrome. Even if anti-adrenergic drugs are used, there is still a sudden death rate of 10% in CPVT, and 50% of patients taking beta-blockers may need ICD implantation.

For patients who experience episodes of ventricular tachycardia during an exercise stress test despite taking beta-blockers, as well as patients who still experience syncope attacks after taking the maximum load of beta-blockers, ICD implantation is recommended. There are also reports on the treatment of CPVT by cardiac sympathectomy.6 For patients experiencing recurrent ICD discharges after ICD implantation, or patients with poor compliance in taking beta-blockers, cardiac sympathectomy may be considered.

The physiological basis for the beneficial effect of cardiac sympathectomomy is as follows: as cardiac sympathectomy can reduce local norepinephrine release by the cardiac sympathetic nerves in the myocardium, malignant ventricular arrhythmias caused by the adrenergic stimulant action may be reduced.

ICD implantation does not reduce the occurrence of catecholaminergic polymorphic ventricular tachycardia events. Appropriate and inappropriate ICD discharges are often caused by exercise, leading to a decline in the quality of life. Moreover, inappropriate discharge will easily lead to the release of endogenous catecholamines, causing a potentially fatal sympathetic electrical storm. For such patients, cardiac sympathectomy can be beneficial.6 Of course, the effectiveness of cardiac sympathectomy still needs to be verified, and this treatment may be considered in patients who experience recurrent ICD discharges despite a loading dose of beta-blocker therapy.

Some studies have shown that SSRIs can reduce the morbidity and mortality of patients with depression after acute coronary syndrome.^2^ Autonomic regulation of cardiac function is usually expressed by non-invasive measurements of heart rate variability (HRV), which is a powerful independent predictor of mortality within the first year after myocardial infarction (MI). It was reported that HRV was significantly lower in coronary disease patients with depression compared with non-depressed patients.^7^,^8^ A study showed that SSRIs facilitated the recovery of HRV after MI.^9^

With depression, chronic depletion of neurotransmitters such as serotonin in the central synaptic clefts could lead to interruption of inhibitory inputs to central sympathetic centres, thereby increasing sympathetic neural discharge. There is increased sympathetic activity in patients with depression. It has been shown that stimulation of central 5-HT receptors can also lead to sympatho-excitation.^9^

In this case, it was found that the patient no longer had ICD discharges after an SSRI was added, possibly indicating that SSRIs may reduce sympathetic activity and have a beneficial therapeutic effect in CPVT. This may provide us with a novel tool to deal with CPVT and improve the quality of patients’ lives.^8^

It was reported that SSRIs had no impact on the release of adrenaline in the hearts of patients with anxiety disorder and panic attacks, but cardiac and whole-body norepinephrine spillover was significantly reduced in those subjects who initially had elevated sympathetic activity.^10^ It was also demonstrated that in such patients with anxiety disorder and near-normal cardiac norepinephrine levels, QT variability was not correlated with cardiac norepinephrine spillover, and SSRI treatment was ineffective.

The findings of Hildreth et al., however, suggested that abnormal serotonergic control of vagal input to the heart might contribute to increased cardiovascular risk.11 The mechanism of anxiety or depression as the trigger(s) for arrhythmias or shock remains unclear.

We suggest that the possible beneficial therapeutic role of serotonin re-uptake inhibitors in the treatment of patients with polymorphic ventricular tachycardia merits further study.
